# Next-Generation Sequencing Approaches for the Identification of Pathognomonic Fusion Transcripts in Sarcomas: The Experience of the Italian ACC Sarcoma Working Group

**DOI:** 10.3389/fonc.2020.00489

**Published:** 2020-04-15

**Authors:** Dominga Racanelli, Monica Brenca, Davide Baldazzi, Frauke Goeman, Beatrice Casini, Biagio De Angelis, Marika Guercio, Giuseppe Maria Milano, Elena Tamborini, Adele Busico, Gianpaolo Dagrada, Cecilia Garofalo, Chiara Caruso, Antonella Brunello, Ymera Pignochino, Enrico Berrino, Giovanni Grignani, Katia Scotlandi, Alessandro Parra, Claudia Maria Hattinger, Toni Ibrahim, Laura Mercatali, Alessandro De Vita, Maria Vincenza Carriero, Matteo Pallocca, Rossella Loria, Renato Covello, Marta Sbaraglia, Angelo Paolo Dei Tos, Rita Falcioni, Roberta Maestro

**Affiliations:** ^1^Unit of Oncogenetics and Functional Oncogenomics, Centro di Riferimento Oncologico di Aviano (CRO Aviano) IRCCS, National Cancer Institute, Aviano, Italy; ^2^Department of Research, Diagnosis and Innovative Technology, IRCCS Regina Elena National Cancer Institute, Rome, Italy; ^3^Department of Onco-Haematology and Cell and Gene Therapy Unit, Bambino Gesù Children's Hospital, IRCCS, Rome, Italy; ^4^Department of Pathology, Fondazione IRCCS Istituto Nazionale dei Tumori, Milan, Italy; ^5^Advanced Translational Research Laboratory, Veneto Institute of Oncology IOV – IRCCS, Padua, Italy; ^6^Medical Oncology 1, Department of Oncology, Veneto Institute of Oncology IOV – IRCCS, Padua, Italy; ^7^Division of Medical Oncology, Candiolo Cancer Institute, FPO-IRCCS, Candiolo, Italy; ^8^Unit of Pathology, Candiolo Cancer Institute FPO-IRCCS, Candiolo, Italy; ^9^Laboratory of Experimental Oncology, IRCCS Istituto Ortopedico Rizzoli, Bologna, Italy; ^10^Osteoncology and Rare Tumors Center, Istituto Scientifico Romagnolo per lo Studio e la Cura dei Tumori (IRST) IRCCS, Meldola, Italy; ^11^Tumor Progression Unit, Department of Experimental Oncology, Istituto Nazionale Tumori Fondazione “G. Pascale” IRCCS, Naples, Italy; ^12^Department of Pathology, Azienda Ospedaliera Universitaria di Padova, Padua, Italy; ^13^Department of Medicine, University of Padua School of Medicine, Padua, Italy

**Keywords:** sarcoma, molecular diagnosis, fusion transcripts, NGS, anchored multiplex PCR, hybrid capture-based panel

## Abstract

This work describes the set-up of a shared platform among the laboratories of the Alleanza Contro il Cancro (ACC) Italian Research Network for the identification of fusion transcripts in sarcomas by using Next Generation Sequencing (NGS). Different NGS approaches, including anchored multiplex PCR and hybrid capture-based panels, were employed to profile a large set of sarcomas of different histotypes. The analysis confirmed the reliability of NGS RNA-based approaches in detecting sarcoma-specific rearrangements. Overall, the anchored multiplex PCR assay proved to be a fast and easy-to-analyze approach for routine diagnostics laboratories.

## Introduction

The term “sarcoma” identifies a heterogeneous group of rare tumors comprising over 60 different histologic variants (1). Due to their rarity and heterogeneity, the accuracy of sarcoma diagnosis remains challenging. In the diagnosis of sarcomas, tumor cell morphology (shape, pattern of growth, microenvironment contexture) and the expression of differentiation markers represent the most important factors, but molecular investigations are increasingly employed to complement these pathological assessments. Indeed, the identification of histotype-specific (pathognomonic) gene alterations is of paramount importance in the differential diagnosis among sarcoma variants, between malignant and benign mimics, as well as between sarcoma and other tumor types (1–3). In particular, about one third of all sarcomas presents pathognomonic chromosome rearrangements (translocations, deletions, insertions) that result in fusion genes and corresponding expression of fusion transcripts (4). Beside diagnostic relevance, the expression of fusion transcripts may have prognostic and/or predictive implications. For example, certain rearrangements, such as those involving *ALK* in inflammatory myofibroblastic tumors or *COL1A1-PDGFB* in dermatofibrosarcoma protuberans, are predictive of the response to tyrosine kinase inhibitors (5, 6). Moreover, the detection of NTRK fusions in a broad range of malignancies, including sarcomas, has gaining much attention due to the recent demonstration of therapeutic efficacy of a new class of tyrosine kinase inhibitors in NTRK rearranged tumors (7–9).

Commonly, FISH or RT-PCR are used to detect fusion events at the genomic or transcriptional level, respectively. However, both methods present limitations. In particular, since they are suited to investigate a specific pre-defined abnormality, they inevitably rely on a prior diagnostic hypothesis (reflex testing). The advent of technologies such as next generation sequencing (NGS), aka massive parallel sequencing, has laid down the bases to overcome this limitation. By allowing the simultaneous analysis of a large set of targets (from few genes to the whole transcriptome/genome) NGS has disclosed the possibility not only to reveal diagnostic/prognostic/predictive genetic abnormalities in the absence of a prior hypothesis but also to identify new aberrations (10–12).

Here we wanted to assess feasibility, reliability, and applicability of NGS-based methods for the detection of sarcoma-associated fusion transcripts in a routine diagnostic setting. Our multicentric analysis confirms the sensitivity of anchored-based NGS profiling approaches and corroborates the suitability of these investigations in the diagnostic setting of sarcomas.

## Materials and Methods

### Case Selection

The study was conducted on a series of 150 sarcoma samples, representative of different sarcoma histotypes, retrieved from the pathological files of the participating institutions (Alleanza Contro il Cancro, ACC, Italian Research Network). Either Formalin-Fixed Paraffin-Embedded (FFPE) or frozen samples were analyzed. All sarcomas included in the study were histopathologically re-evaluated on hematoxylin-eosin stained slides, and representative areas were selected for molecular analyses.

### NGS-based Fusion Transcript Identification

RNA was extracted from 5 to 10 μm-FFPE tissue sections using the Qiagen miRNeasy FFPE kit (Qiagen, Valencia, CA, USA) or the Invitrogen RecoverAll Total Nucleic Acid Isolation kit (Thermo Fisher Scientific, Waltham, MA, USA). For frozen samples the TRIzol reagent (Life Technologies Italia, Monza, Italy) followed by the RNeasy MinElute cleanup (Qiagen, Valencia, CA, USA) was used. Total RNA was quantified by using a Qubit fluorometer (Thermo Fisher Scientific, Waltham, MA, USA). Quality was checked with the RNA 6000 Nano Kit on a 2100 Bioanalyzer (Agilent Technologies, Santa Clara, CA, USA), or by using the Archer PreSeq^TM^ RNA QC qPCR Assay (ArcherDX, Boulder, CO, USA) and a threshold of DV_200_ >30 or PreSeq Cq <31 was used to identify high quality RNA, respectively.

FISH, RT-PCR, RT-qPCR, and IHC, used as primary detection approaches for the detection of possible fusion events, were performed during routine diagnostic procedures according to laboratory standard guidelines and validated reagents.

Three different commercially available NGS-based fusion panels were selected based on their capacity to cover most genes known to be involved in sarcoma-relevant fusions: an anchored multiplex PCR-based assay, namely the Archer FusionPlex Sarcoma kit (AMP-FPS)(ArcherDX, Boulder, CO, USA), covering 26 genes involved in sarcoma-associated fusions; two hybrid capture-based (HC) assays, namely the TruSight RNA Fusion Panel (TS-Fusion) (Illumina Inc., San Diego, CA, USA) and the TruSight RNA PanCancer Panel (TS-PanCancer) (Illumina Inc., San Diego, CA, USA) covering 507 and 1,385 genes commonly involved in cancer, respectively. Both HC assays included the 26 genes covered by the AMP-FPS kit. In a subset of samples, a customized version of the AMP-FPS panel was used to detect PAX3 fusion transcripts. Specifically, the assay was integrated with PAX3-specific primers (exons 6, 7 and 8) designed by using the Archer Assay Designer tool (ArcherDX, Boulder, CO, USA).

Libraries for all three panels were prepared and checked for quality according to the manufacturer's instructions, starting from 100 to 250 ng of RNA as input.

AMP-FPS libraries were run on either Illumina (MiSeq or NextSeq 500 Illumina Inc., San Diego, CA, USA) or Thermo (Ion S5 Thermo Fisher Scientific, Waltham, MA, USA) sequencing platforms, according to the manufacturer's instructions. HC-based libraries were sequenced on Illumina MiSeq instruments. Illumina TS-Fusion and TS-PanCancer sequencing data were analyzed by using the dedicated Illumina BaseSpace RNA-Seq Alignment tool (v.s.2.0.2), which relies on STAR and Manta algorithms (13, 14). PAR-masked/(RefSeq)hg19 was used as reference genome. A minimum of 3 million reads was obtained per sample (range 3007307–6284475). The mean percentage of reads aligned to the human genome was 98.9% (range 96.4–99.7%); the mean proportion of reads aligned to ribosomal RNA was below 2% (range 0.2–6.1%) and mean insert size was 134 bp (range 107–155 bp), in line with literature data (15). Only high-confidence fusions that passed default thresholds of the RNA-Seq Alignment tool (PASS) were recorded.

The Archer Analysis suite (v 5.1 or v 6.0) was exploited for the analysis of AMP-FPS panel results, using default settings. Default parameters (QC PASS) that, according to the Archer user manual, allow to achieve up to 95% of sensitivity in fusion detection, were employed to assess data quality. Samples included in the study met the quality cutoffs set by the Archer Analysis platform but in a few cases that, although not fulfilling all default criteria, nevertheless yielded high confidence fusion calls (cases #9, 31, 37, 47, 57, 60, 80, 126). Fusions were recorded as “high confidence calls”(strong = true in output table) if they passed all “strong evidence” default filters as described in the Archer analysis user manual (briefly: breakpoint spanning reads that support the candidate ≥ 5; “fusion_percent_of_GSP2_reads”, i.e., proportion of breakpoint spanning reads that support the candidate relative to the total number of reads spanning the breakpoint ≥10%; “min_unique_start_sites_for_strong_fusion” ≥3; fusion recorded in the Quiver database or not fulfilling the “negative evidence criteria”).

Of 48 cases (12 of the first set and 36 of the second set) where a fusion was detected by NGS but the partner genes had not been previously determined by the primary detection method, material was available for orthogonal validations (RT-PCR) in 39 cases, confirming NGS results. The involvement of *SSX4 (SS18-SSX4)*, called sometime by the AMP-FPS assay in synovial sarcoma samples, was checked by nested RT-PCR (primers: Fw-SS18 GGACCACCACAGCCACCCCA, Rev-SSX ATGTTTCCCCCTTTTGGGTC; Rev-SSX4 GTCTTGTTAATCTTCTCCAAGG) and Sanger sequencing on a single index case.

For second level bioinformatic analyses of HC library raw data, Arriba, STAR-Fusion and Pizzly (16–18), administered through a command line interface, were employed for fusion calling using default settings.

## Results

### NGS-based Identification of Fusion Transcripts: Panel Comparison

As a first step toward the assessment of suitability of NGS-based approaches for the detection of pathognomonic fusions in sarcomas, performance and ease-of-use (library preparation complexity, hands-on time, user-friendly dedicated bioinformatic analysis tool) of three different NGS fusion panels were evaluated on a set of sarcoma samples previously characterized by either FISH or RT-qPCR for gene fusions (Table 1). Twenty-six samples were analyzed with a hybrid capture-based panel (HC) (Illumina TS-Fusion). Twenty samples were analyzed with an anchored multiplex PCR panel (Archer AMP-FPS), 19 of which investigated also with the Illumina TS-Fusion. In addition, 9 samples were profiled with a more comprehensive HC panel (Illumina TS-PanCancer).

**Table 1:** NGS fusion profiling: panel comparison.

**Nr**	**Diagnosis**	**Pre-detected genetic abnormality**	**Primary detection method**	**Histotype-specific fusion detected by the indicated NGS approach**			**Other passing filters fusions (assay detecting the additional fusion)**
				**AMP-FPS**	**TS-Fusion**	**TS-PanCancer**	
1	Dermatofibrosarcoma Protuberans	*PDGFB*	FISH	*COL1A1-PDGFB^*IL*^*	*COL1A1-PDGFB*	*COL1A1-PDGFB*	NFD
2	Ewing Sarcoma	*EWSR1*	FISH	*EWSR1-FLI1^*IL*^*	*EWSR1-FLI1*	*EWSR1-FLI1*	NFD
3	Infantile Fibrosarcoma	*ETV6*	FISH	*ETV6-NTRK3^*IL*^*	*ETV6-NTRK3*	*ETV6-NTRK3*	NFD
4	Synovial Sarcoma	*SS18-SSX1*	RT-qPCR	*SS18-SSX1^*IL*^*	*SS18-SSX1*	*SS18-SSX1*	*SS18-SSX4* (AMP-FPS^IL^)
5	Synovial Sarcoma	*SS18*	FISH	*SS18-SSX2^*IL*^*	*SS18-SSX2*	*SS18-SSX2*	*SS18-SSX4* (AMP-FPS^IL^)
6	Myoepithelioma (soft tissue)	*EWSR1*	FISH	*EWSR1-ATF1^*IL*^*	*EWSR1-ATF1*	NFD	*ATF1-EWSR1* (TS-Fusion,TS-PanCancer)
7	Extraskeletal Myxoid Chondrosarcoma	*EWSR1-NR4A3*	RT-qPCR	*EWSR1-NR4A3^*IL*^*	NFD	NFD	NFD
8	Clear Cell sarcoma	*EWSR1*	FISH	*EWSR1-ATF1^*T, IL*^*	NFD	nd	NFD
9	Ewing Sarcoma	*EWSR1-FLI1*	RT-qPCR	*EWSR1-FLI1^*T, IL*^*	*EWSR1-FLI1*	nd	NFD
10	Ewing Sarcoma	*EWSR1-FLI1*	RT-qPCR	*EWSR1-FLI1^*T, IL*^*	*EWSR1-FLI1*	nd	NFD
11	Ewing Sarcoma	*EWSR1-ERG*	RT-qPCR	*EWSR1-ERG^*T, IL*^*	*EWSR1-ERG*	nd	*EWSR1-ERG-EWSR1* (AMP-FPS^IL^)
12	Extraskeletal Myxoid Chondrosarcoma	*EWSR1-NR4A3*	RT-qPCR	*EWSR1-NR4A3^*T*^*	*EWSR1-NR4A3*	nd	NFD
13	Myxoid Liposarcoma	*FUS-DDIT3*	RT-qPCR	*FUS-DDIT3^*IL*^*	*FUS-DDIT3*	nd	NFD
14	Myxoid Liposarcoma	*FUS-DDIT3*	RT-qPCR	*FUS-DDIT3^*T, IL*^*	*FUS-DDIT3*	nd	*DDIT3-FUS* (TS-Fusion)
15	Myxoid Liposarcoma	*FUS-DDIT3*	RT-qPCR	*FUS-DDIT3^*T, IL*^*	*FUS-DDIT3*	nd	*FUS-DDIT3-DLG2* (AMP-FPS^IL^)
16	Synovial Sarcoma	*SS18-SSX1*	RT-qPCR	*SS18-SSX1^*IL*^*	*SS18-SSX1*	nd	*SS18-SSX4-SS18; SS18-SSX4* (AMP-FPS^IL^)
17	Synovial Sarcoma	*SS18*	FISH	*SS18-SSX1^*IL*^*	*SS18-SSX1*	nd	NFD
18	Synovial Sarcoma	*SS18-SSX1*	RT-qPCR	*SS18-SSX1^*IL*^*	*SS18-SSX1*	nd	*SS18-SSX4* (AMP-FPS^IL^)
19	Synovial Sarcoma	*SS18-SSX1*	RT-qPCR	*SS18-SSX1^*T, IL*^*	*SS18-SSX1*	nd	*SS18-SSX1/4-SS18; SS18-SSX4* (AMP-FPS^IL^)
20	Myxoid Liposarcoma	*DDIT3*	FISH	*FUS-DDIT3^*IL*^*	nd	*FUS-DDIT3*	*DDIT3-FUS* (TS-PanCancer)
21	Myxoid Liposarcoma	*DDIT3*	FISH	nd	FUS-DDIT3	NFD	NFD
22	Synovial Sarcoma	*SS18*	FISH	nd	*SS18-SSX1*	nd	NFD
23	Synovial Sarcoma	*SS18*	FISH	nd	*SS18-SSX1*	nd	NFD
24	Myxoid Fibrosarcoma	*FUS*	FISH	nd	*FUS-CREB3L2*	nd	NFD
25	Myxoid Liposarcoma	*FUS-DDIT3*	RT-qPCR	nd	*FUS-DDIT3*	nd	*DDIT3-FUS* (TS-Fusion)
26	Myxoid Liposarcoma	*DDIT3*	FISH	nd	NFD	nd	NFD
27	Undifferentiated Round Cell, Ewing-Like Sarcoma	*CIC*	FISH	nd	NFD	nd	NFD

*NFD, no histotype-specific fusion detected; nd, not done; FISH, fluorescent in situ hybridization; RT-qPCR, reverse transcriptase- quantitative PCR; Sequencing platform used: T, Thermo platform; IL, Illumina platform*.

All three targeted RNA-sequencing panels permit the identification of common and known fusions involved in sarcomas, but also the discovery of novel fusions. The AMP-FPS panel targets a limited set of genes (26 target genes) that are commonly involved in sarcoma-associated fusions. This AMP-FPS panel employs unidirectional gene-specific primers to detect fusion transcripts involving target genes. In addition, molecular barcodes are included to enable single molecule counting, de-duplication and error correction, thus allowing quantitative analysis and confident mutation calling.

In HC-based panels the transcripts of interest are enriched by hybridization and capture with biotinylated probes (507 genes in TS-Fusion, 1385 genes in TS-PanCancer, in both cases including the 26 genes targeted by the AMP-FPS panel).

Raw data obtained with the different panels were then analyzed using the dedicated bioinformatic suite (BaseSpace RNA-Seq Alignment for Illumina HC panels, Archer Analysis platform for the AMP-FPS panel). The AMP-FPS assay correctly identified the pathognomonic fusion in all samples analyzed (20/20), irrespective of the sequencing platform used (Thermo and/or Illumina), demonstrating an excellent sensitivity. The pathognomonic fusion was correctly called in 22/26 samples analyzed with the TS-Fusion HC assay. Of the 9 cases analyzed with the TS-PanCancer HC panel, the dedicated bioinformatic tool identified the diagnostic fusion in 7 cases, in one of these as a reciprocal fusion. To further explore the performance of HC panels, data generated with TS-Fusion and TS-PanCancer panels were re-evaluated with additional algorithms, namely Arriba, STAR-Fusion and Pizzly (16–18). Although impractical in a routine diagnostic setting, as they rely on a command line interface, these tools are reported to have high fusion detection rates (16–18). With the exception of case #27, for which no algorithm detected, as high confidence calls, fusions involving the *CIC* gene, apparently rearranged according to FISH, at least one fusion caller was capable of detecting, among others, a fusion transcript involving the target gene in cases previously scored negative with the BaseSpace RNA-Seq Alignment tool, emphasizing the importance of software sensitivity in data analysis (Supplemental Tables 1–3).

Additional passing filters fusions (in frame and out of frame) were occasionally called beside the pathognomonic one, but the actual biological significance of these alterations is unclear. For instance, beside the canonical fusion involving *SS18* and *SSX1* or *SSX2*, additional fusions involving *SSX4* were called in 5/6 synovial sarcomas analyzed with the AMP-FPS panel. It should be pointed out that the AMP-FPS approach relies on relatively small amplicons. Thus, in the presence of highly homologous genes (e.g., *SSX1, SSX2, SSX4*), this technique may fail to properly distinguish the target (19). Indeed, a deeper analysis of an index case confirmed the expression of *SS18-SSX1*, suggesting that the alleged *SS18-SSX4* fusion was likely an alignment artifact.

**Figure 1:**
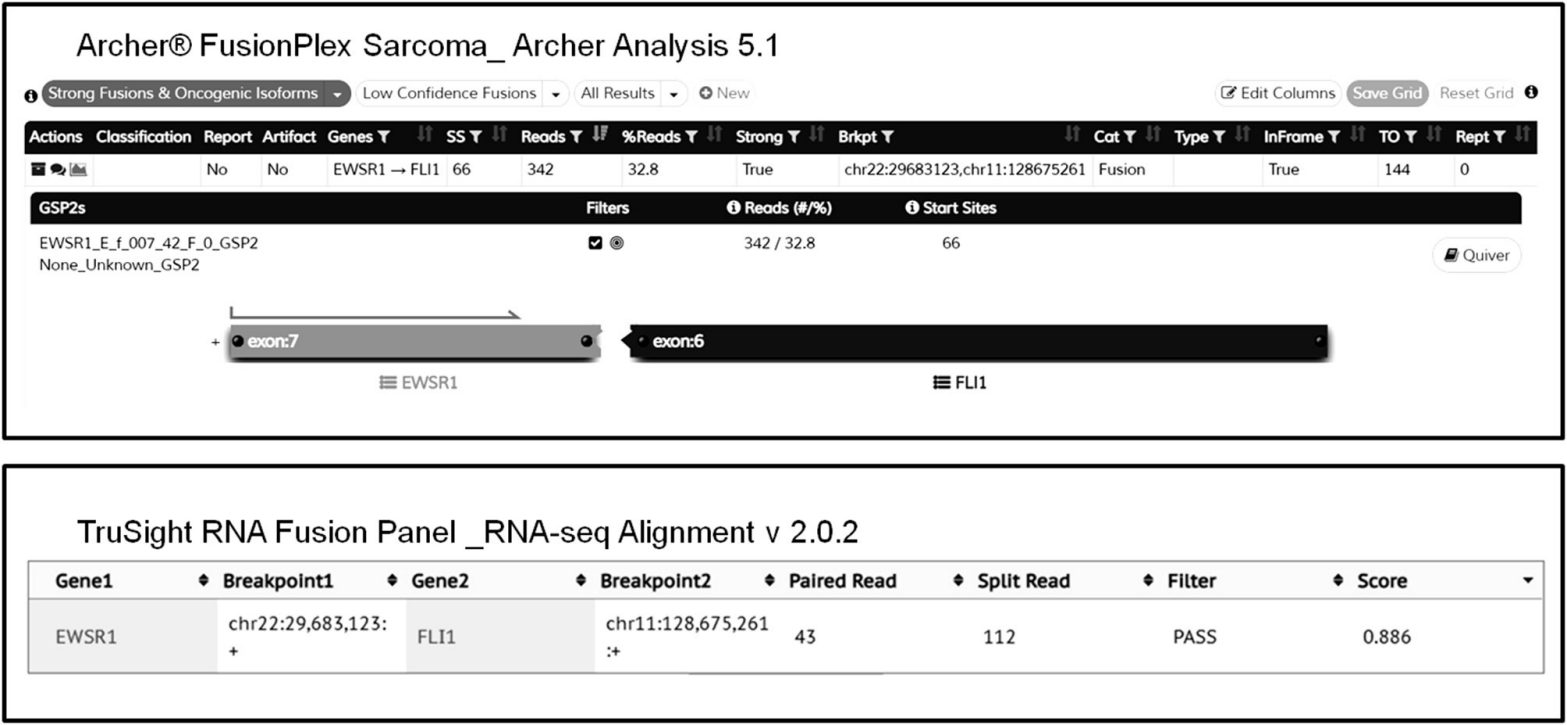
Representative graphical output of Archer Analysis (top) and Illumina BaseSpace RNA-Seq Alignment (bottom) tools. The EWSR1-FLI1 fusion detected in sample #2 by both AMP-FPS and HC panels is shown.

Overall, both AMP-FPS and HC assays demonstrated a good detection capability. The HC assays were definitively more comprehensive and suitable for a research environment. In contrast, the AMP-FPS panel was limited in breath (only 26 target genes), and hence with reduced capacity of discovering new fusions, but definitively provided for a better ease-of-use. In particular, the hands-on-time for library preparation was reduced. Moreover, compared to the BaseSpace RNA-Seq Alignment, the AMP-FPS dedicated bioinformatic analysis tool (Archer Analysis platform) featured a more user-friendly graphical interface with detailed and straightforward information about the fusion (exons involved, in frame/out of frame, confidence of the call) (Figure 1).

On the whole, we considered the AMP-FPS assay more suitable for routine diagnostics.

### Validation on a Larger Set of Cases of the AMP-FPS Fusion Transcript Assay

Based on these results, with a view to translating NGS-based fusion identification in a routine diagnostic setting, we sought to extend the evaluation of the AMP-FPS panel (on either a Thermo or an Illumina sequencing platform) to 123 additional cases (Table 2).

**Table 2:** Validation of the AMP-FPS fusion transcript assay.

**Nr**	**Diagnosis**	**Pre-detected genetic abnormality**	**Primary detection method**	**Sequencing platfom**	**Histotype-specific fusion detected**	**Other passing filters fusions**
28	Askin Tumor	*EWSR1-ERG*	RT-qPCR	Illumina	*EWSR1-ERG*	*EWSR1-unl-ERG*
29	Congenital Fibrosarcoma	*ETV6-NTRK3*	RT-qPCR	Illumina	*ETV6-NTRK3*	NFD
30	Dermatofibrosarcoma Protuberans	*COL1A1-PDGFB*	FISH	Thermo	*COL1A1-PDGFB*	NFD
31	Dermatofibrosarcoma Protuberans	*COL1A1-PDGFB*	RT-qPCR	Illumina	*COL1A1-PDGFB*	NFD
32	Ewing Sarcoma	*EWSR1*	FISH	Thermo	*EWSR-FLI1*	NFD
33	Ewing Sarcoma	*EWSR1*	FISH	Thermo	*EWSR-FLI1*	NFD
34	Ewing Sarcoma	*EWSR1*	FISH	Thermo	*EWSR1-PATZ1*	NFD
35	Ewing Sarcoma	*EWSR1*	FISH	Thermo	*EWSR-FLI1*	NFD
36	Ewing Sarcoma	*EWSR1*	FISH	Thermo	*EWSR-FLI1*	NFD
37	Ewing Sarcoma	*EWSR1-FLI1*	RT-qPCR	Illumina	*EWSR1-FLI1*	*FXR2-CAMTA1*
38	Ewing Sarcoma	*EWSR1-FLI1*	RT-qPCR	Illumina	*EWSR1-FLI1*	NFD
39	Ewing Sarcoma	*EWSR1-FLI1*	RT-qPCR	Illumina	*EWSR1-FLI1*	NFD
40	Ewing Sarcoma	*EWSR1-ERG*	RT-qPCR	Illumina	*EWSR1-ERG*	*EWSR1-unl-EWSR1-ERG; FUS-ERG; EWSR1-ERG-EWSR1;*
41	Ewing Sarcoma	*EWSR1-FLI1*	FISH	Illumina	*EWSR1-FLI1*	*EWSR1-FLI1-EWSR1*
42	Ewing Sarcoma	*EWSR1*	FISH	Thermo	*EWSR1-FLI1*	NFD
43	Ewing Sarcoma	*EWSR1-FLI1*	RT-qPCR	Thermo	*EWSR1-FLI1*	NFD
44	Ewing Sarcoma	*EWSR1-FLI1*	RT-qPCR	Thermo	*EWSR1-FLI1*	NFD
45	Ewing Sarcoma	*EWSR1-FLI1*	RT-qPCR	Thermo	*EWSR1-FLI1*	NFD
46	Ewing Sarcoma	*EWSR1-FLI1*	RT-qPCR	Thermo	*EWSR1-FLI1*	NFD
47	Ewing Sarcoma	*EWSR1-FLI1*	RT-qPCR	Thermo	*EWSR1-FLI1*	NFD
48	Ewing Sarcoma	*EWSR1-FLI1*	RT-qPCR	Thermo	*EWSR1-FLI1*	NFD
49	Ewing Sarcoma	*EWSR1-FLI1*	RT-qPCR	Thermo	*EWSR1-FLI1*	NFD
50	Ewing Sarcoma	*EWSR1-FLI1*	RT-qPCR	Illumina	*EWSR1-FLI1*	NFD
51	Ewing Sarcoma	*EWSR1*	FISH	Illumina	*EWSR1-FLI1*	NFD
52	Ewing Sarcoma	*FUS*	FISH	Thermo	*FUS-ERG*	NFD
53	Ewing-like Sarcoma	*BCOR-CCNB3*	RT-qPCR	Illumina	*BCOR-CCNB3*	NFD
54	Ewing-like Sarcoma	*CIC-DUX4*	RT-qPCR	Illumina	*CIC-DUX4*	NFD
55	Extraskeletal Myxoid Chondrosarcoma	*NR4A3*	FISH	Illumina	*EWSR1-NR4A3*	NFD
56	Extraskeletal Myxoid Chondrosarcoma	*EWSR1*	FISH	Illumina	*EWSR1-NR4A3*	NFD
57	Extraskeletal Myxoid Chondrosarcoma	*EWSR1-NR4A3*	RT-qPCR	Illumina	*EWSR1-NR4A3*	NFD
58	Extraskeletal Myxoid Chondrosarcoma	*TAF15-NR4A3*	RT-qPCR	Illumina	*TAF15-NR4A3*	NFD
59	Extraskeletal Myxoid Chondrosarcoma	*EWSR1-NR4A3*	RT-qPCR	Illumina	*EWSR1-NR4A3*	NFD
60	Extraskeletal Myxoid Chondrosarcoma	*EWSR1-NR4A3*	RT-qPCR	Illumina	*EWSR1-NR4A3*	NFD
61	Extraskeletal Myxoid Chondrosarcoma	*EWSR1-NR4A3*	RT-qPCR	Illumina	*EWSR1-NR4A3*	NFD
62	Extraskeletal Myxoid Chondrosarcoma	*EWSR1-NR4A3*	RT-qPCR	Illumina	*EWSR1-NR4A3*	NFD
63	Extraskeletal Myxoid Chondrosarcoma	*EWSR1-NR4A3*	RT-qPCR	Illumina	*EWSR1-NR4A3*	NFD
64	Extraskeletal Myxoid Chondrosarcoma	*NR4A3*	FISH	Illumina	*EWSR1-NR4A3*	NFD
65	Extraskeletal Myxoid Chondrosarcoma	*EWSR1-NR4A3*	RT-qPCR	Illumina	*EWSR1-NR4A3*	NFD
66	Myoepitelial carcinoma (soft tissue)	*EWSR1*	FISH	Illumina	*EWSR1-ATF1*	NFD
67	Myoepithelioma (soft tissue)	*EWSR1*	FISH	Illumina	*EWSR1-ATF1*	NFD
68	Myxoid Liposarcoma	*FUS-DDIT3*	RT-PCR	Thermo	*FUS-DDIT3*	NFD
69	Myxoid Liposarcoma	*FUS-DDIT3*	RT-qPCR	Illumina	*FUS-DDIT3*	NFD
70	Myxoid Liposarcoma	*FUS-DDIT3*	FISH	Thermo	*FUS-DDIT3*	NFD
71	Myxoid Liposarcoma	*FUS-DDIT3*	FISH	Illumina	*FUS-DDIT3*	NFD
72	Myxoid Liposarcoma	*FUS-DDIT3*	FISH	Illumina	*FUS-DDIT3*	NFD
73	Nodular Fascitis	*USP6*	FISH	Thermo	*MYH9-USP6*	NFD
74	Rhabdomyosarcoma, alveolar	*PAX3-FOXO1*	RT-PCR	Thermo	*PAX3-FOXO1*	NFD
75	Rhabdomyosarcoma, alveolar	*PAX3-FOXO1*	RT-PCR	Thermo	*PAX3-FOXO1*	NFD
76	Rhabdomyosarcoma, alveolar	*PAX3-FOXO1*	RT-PCR	Thermo	*PAX3-FOXO1*	NFD
77	Rhabdomyosarcoma, alveolar	*PAX3-FOXO1*	RT-qPCR	Illumina	*PAX3-FOXO1*	NFD
78	Rhabdomyosarcoma, alveolar	*PAX3-FOXO1*	RT-qPCR	Illumina	*PAX3-FOXO1*	NFD
79	Rhabdomyosarcoma, alveolar	*PAX3-FOXO1*	RT-qPCR	Illumina	*PAX3-FOXO1*	NFD
80	Rhabdomyosarcoma, alveolar	*PAX3-FOXO1*	RT-qPCR	Illumina	*PAX3-FOXO1*	NFD
81	Rhabdomyosarcoma, alveolar	*PAX3-FOXO1*	RT-qPCR	Illumina	*PAX3 - FOXO1*	*FOXO1-PAX3*
82	Rhabdomyosarcoma, alveolar	*PAX3-FOXO1*	RT-qPCR	Illumina	*PAX3-FOXO1*	NFD
83	Rhabdomyosarcoma, splindle cell	*SRF-NCOA2*	RT-qPCR	Illumina	*SRF- NCOA2*	NFD
84	Sarcoma NOS	*EWSR1*	FISH	Illumina	*EWSR1-FLI1*	NFD
85	Solitary Fibrous Tumor	*STAT6*	IHC	Thermo	*NAB2-STAT6*	NFD
86	Synovial Sarcoma	*SS18-SSX2*	RT-qPCR	Illumina	*SS18-SSX2*	*SS18-SSX4;SS18-SSX1; complex SS18-SSX2 fusions*
87	Synovial Sarcoma	*SS18*	FISH	Illumina	*SS18-SSX1*	*SS18-SSX4; SS18-SSX4-SS18*
88	Synovial Sarcoma	*SS18*	FISH	Thermo	*SS18-SSX1*	NFD
89	Synovial Sarcoma	*SS18-SSX1*	RT-qPCR	Illumina	*SS18-SSX1*	NFD
90	Synovial Sarcoma	*SS18-SSX1*	RT-qPCR	Thermo	*SS18-SSX1*	NFD
91	Synovial Sarcoma	*SS18-SSX1*	RT-qPCR	Thermo	*SS18-SSX1*	*SS18-SSX2*
92	Synovial Sarcoma	*SS18-SSX1*	RT-qPCR	Thermo	*SS18-SSX1*	*SS18-SSX4*
93	Synovial Sarcoma	*SS18-SSX1*	RT-qPCR	Thermo	*SS18-SSX1*	*SS18-SSX4*
94	Synovial Sarcoma	*SS18*	FISH	Illumina	*SS18-SSX1*	*SS18-SSX4-SS18*
95	Synovial Sarcoma	*SS18-SSX2*	RT-qPCR	Illumina	*SS18-SSX2*	NFD
96	Synovial Sarcoma	*SS18*	FISH	Illumina	*SS18-SSX1*	*SS18-SSX4*
97	Synovial Sarcoma	*SS18-SSX1*	RT-qPCR	Thermo	*SS18-SSX1*	*SS18-SSX4*
98	Clear Cell Sarcoma	*EWSR1*	FISH	Thermo	*EWSR1-CREB1*	NFD
99	Endometrial Stromal Sarcoma	*BCOR*	FISH	Thermo	NFD	NFD
100	Extraskeletal Myxoid Chondrosarcoma	*NR4A3*	FISH	Illumina	NFD	NFD
101	Myoepithelioma (soft tissue)	*EWSR1*	FISH	Illumina	NFD	NFD
102	Myxoid Fibrosarcoma	*FUS*	FISH	Illumina	NFD	NFD
103	Myxoid Liposarcoma	*DDIT3*	FISH	Illumina	NFD	NFD
104	Nodular Fasciitis	*USP6*	FISH	Thermo	NFD	NFD
105	Rhabdomyosarcoma, alveolar	*FOXO1*	FISH	Thermo	NFD	NFD
106	Sarcoma NOS	*BCOR*	FISH	Thermo	NFD	NFD
107	Solitary Fibrous Tumor	*EWSR1*	FISH	Illumina	NFD	NFD
108	Undifferentiated round cell, Ewing-Like Sarcoma	*CIC*	FISH	Illumina	NFD	NFD
109	Lipoblastoma	*PLAG1 neg*	FISH	Illumina	NFD	NFD
110	Myxoid Fibrosarcoma	*EWSR1, FUS neg*	FISH	Thermo	NFD	NFD
111	Myxoid Fibrosarcoma	*EWSR1, FUS neg*	FISH	Thermo	NFD	NFD
112	Myxoid Fibrosarcoma	12q13-15 amp	FISH	Thermo	NFD	NFD
113	Rhabdomyosarcoma, alveolar	*FOXO1 neg*	FISH	Thermo	NFD	NFD
114	Rhabdomyosarcoma, embryonal	*FOXO1 neg*	FISH	Illumina	NFD	NFD
115	Rhabdomyosarcoma, embryonal	*FOXO1 neg*	FISH	Illumina	NFD	NFD
116	Rhabdomyosarcoma, embryonal	*FOXO1 neg*	FISH	Illumina	NFD	NFD
117	Sarcoma NOS	*EWSR1 neg*	FISH	Illumina	*CIC-DUX4*	NFD
118	Small Round Cell Tumor	*EWSR1, BCOR, FUS, CIC neg*	FISH	Thermo	NFD	NFD
119	Undifferentiated Sarcoma	*EWSR1 neg*	FISH	Illumina	*CIC-DUX4*	NFD
120	Undifferentiated Sarcoma	12q13-15 amp	FISH	Thermo	NFD	NFD
121	Undifferentiated Sarcoma	12q13-15 amp	FISH	Thermo	NFD	*HMGA2-LGR5*
122	Biphenotypic Sinonasal Sarcoma	nd	nd	Thermo	*PAX3-MAML3^§^*	NFD
123	Biphenotypic Sinonasal Sarcoma	nd	nd	Thermo	*PAX3-MAML3^§^*	NFD
124	Biphenotypic Sinonasal Sarcoma	nd	nd	Thermo	*PAX3-MAML3^§^*	NFD
125	Dermatofibrosarcoma Protuberans	nd	nd	Thermo	*COL1A1-PDGFB*	NFD
126	Endometrial Stromal Sarcoma	nd	nd	Thermo	*YWHAE-NUTM2B*	NFD
127	Gastrointestinal Neuroectodermal Tumor	nd	nd	Thermo	*EWSR1-CREB1*	*SS18-PTRF*
128	Inflammatory Myofibroblastic Sarcoma	nd	nd	Illumina	*TPM4-ALK*	NFD
129	Inflammatory Myofibroblastic Tumor	nd	nd	Thermo	*TFG-ROS1*	NFD
130	Myoepithelioma (bone)	nd	nd	Illumina	*FUS-NFATC2*	NFD
131	Myoepithelioma (soft tissue)	nd	nd	Illumina	*TRPS1-PLAG1*	NFD
132	Sclerosing Epitheliodid Fibrosarcoma	nd	nd	Illumina	*EWSR1-CREB3L2*	NFD
133	Sclerosing epitheliodid fibrosarcoma (soft tissue)	nd	nd	Illumina	*FUS-CREB3L2*	NFD
134	Solitary Fibrous Tumor	nd	nd	Thermo	*NAB2-STAT6*	NFD
135	Chondrosarcoma	nd	nd	Thermo	NFD	NFD
136	Endometrial Stromal Sarcoma	nd	nd	Thermo	NFD	NFD
137	Epithelioid Angiosarcoma	nd	nd	Illumina	NFD	NFD
138	Follicular Dendritic Cell Sarcoma	nd	nd	Thermo	NFD	NFD
139	Leiomyosarcoma	nd	nd	Illumina	NFD	NFD
140	Leiomyosarcoma	nd	nd	Thermo	NFD	NFD
141	Myoepithelioma (bone)	nd	nd	Illumina	NFD	NFD
142	Myxoid Fibrosarcoma	nd	nd	Thermo	NFD	NFD
143	Myxoinflammatory Fibroblastic Sarcoma	nd	nd	Illumina	NFD	NFD
144	Osteosarcoma	nd	nd	Illumina	NFD	NFD
145	Osteosarcoma	nd	nd	Illumina	NFD	NFD
146	Pleomophic Sarcoma	nd	nd	Thermo	NFD	NFD
147	Pleomophic Sarcoma	nd	nd	Thermo	NFD	NFD
148	Pleomophic Sarcoma	nd	nd	Thermo	NFD	NFD
149	Sarcoma NOS HG Myxoid	nd	FISH	Thermo	NFD	NFD
150	Undifferentiated Sarcoma	nd	nd	Illumina	NFD	NFD

*NFD, no histotype-specific fusion detected; nd, not done; amp, amplification; neg, negative; RT-PCR, reverse transcriptase-PCR; FISH, fluorescent in situ hybridization; RT-qPCR, reverse transcriptase-quantitative PCR; IHC, immunohistochemistry; unl, unaligned sequence. PAX3-MAML3§: fusion detected with a PAX3-customized AMP-FPS Panel. This sample scored negative with the standard AMP-FPS Panel*.

Overall, the AMP-FPS panel confirmed the good performance. Of 81 cases with a pre-detected genetic abnormality suggestive of a fusion event, this NGS assay proved effective in 71, with orthogonal validations (RT-PCR) confirming the NGS result where appropriate (see Material and Methods). In the remaining 10 cases, a gene rearrangement was suggested by FISH. Nevertheless, although samples passed quality filters, the AMP-FPS assay failed to detect a fusion transcript. There are several possible explanations for this discrepancy including inadequate tumor cell fraction or low expression levels of the fusion transcript, chromosome rearrangements not yielding a fusion transcript, unusual breakpoints not covered by the assay or lack of primers covering the target gene. For instance, in two tumors (one endometrial stromal sarcoma and one sarcoma NOS) FISH indicated a rearrangement of the *BCOR* gene with an unknown partner. It is worth noting that the commercial AMP-FPS panel used in this study does not include primers for *BCOR*. Moreover, beside the common *CCNB3* partner (covered by the panel), *BCOR* has been reported to fuse with other genes which are also not targeted by the AMP-FPS assay (e.g., *ZC3H7B, MAML3, CIITA*) (20–23). Thus, in the absence of probes for *BCOR* and potential partner genes, the failure of the assay in the 2 *BCOR* rearranged tumors of our series is not surprising. The same holds true for rearrangements involving *NR4A3* in extraskeletal myxoid chondrosarcomas: while the AMP-FPS assay covers the most *NR4A3* common partners (*EWSR1, TAF15, TCF12, TFG*) it lacks probes for both *NR4A3* and uncommon partners (24), thus scoring negative in the presence of alternative fusions.

**Figure 2:**
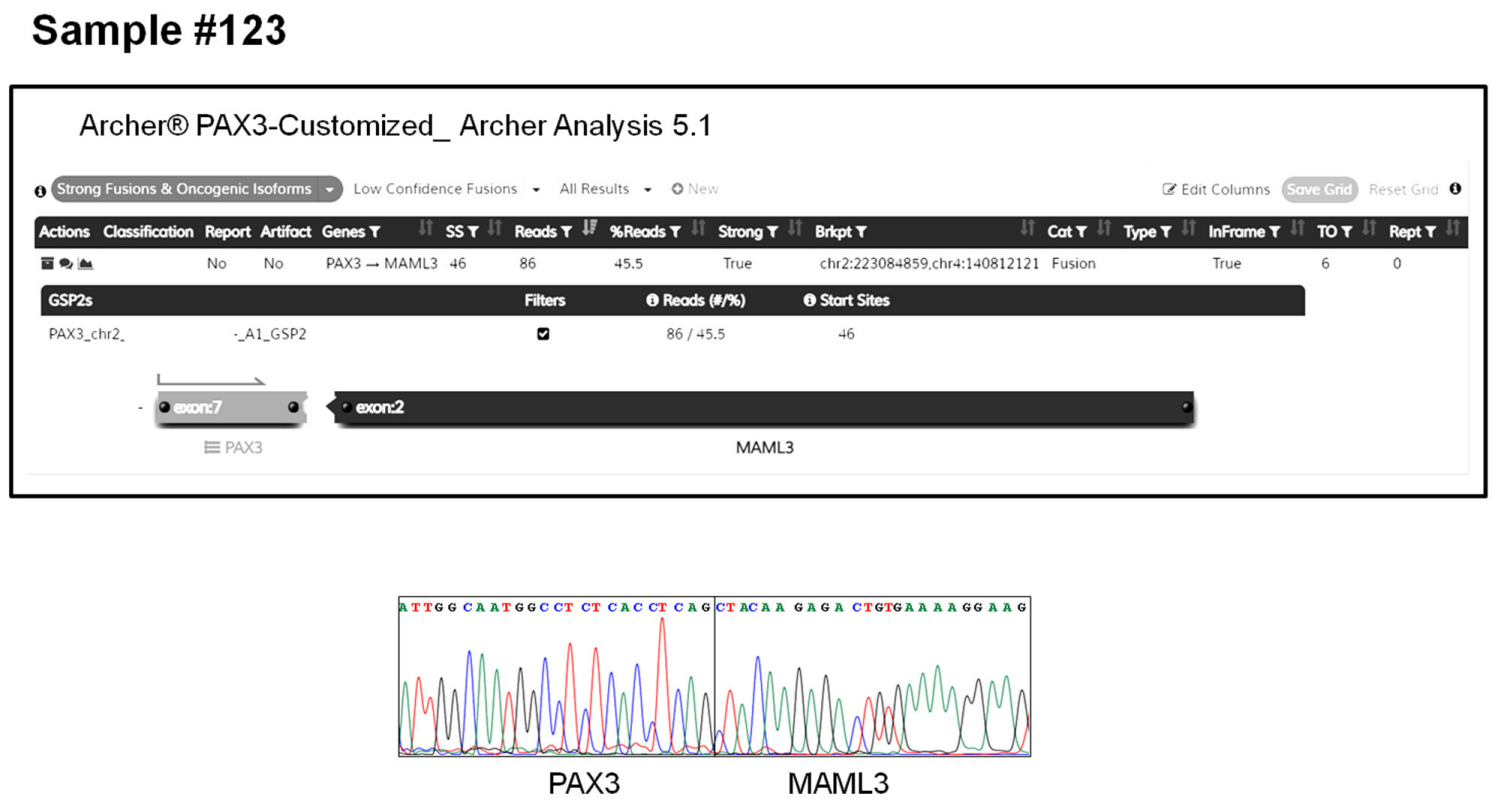
PAX3-MAML3 fusion detected by the customized AMP-FPS panel in a representative case of biphenotypic sinonasal sarcoma (sample #123). The top panel shows the output of the Archer Analysis tool. The bottom panel shows the validation of the fusion by RT-PCR sequencing.

The AMP-FPS assay failed to detect any fusion also in 3 cases of biphenotypic sinonasal sarcoma. Although in these cases no prior investigation (FISH or RT-PCR) was performed, this tumor is known to be typified by gene fusions involving the *PAX3* gene (25). Since the *PAX3* gene is not covered by the commercial AMP-FPS panel, we commissioned a customization of the assay by spiking-in primers to cover *PAX3* fusions. By using this customized AMP-FPS assay we were able to demonstrate and validate that all 3 cases expressed a *PAX3-MAML3* chimeric transcript (Figure 2).

Interestingly, a rare EWSR1-PATZ1 fusion was detected by AMP-FPS in one EWSR1 FISH-positive Ewing sarcoma (case #34). This fusion had been previously described in rare cases of spindled or small round cell sarcomas and it is considered to identify a distinct, Ewing-like entity (26). Moreover, the NGS profiling allowed the detection of disease-associated fusion transcripts also in a set of cases for which no prior molecular data was available or scored negative for FISH. These included one dermatofibrosarcoma protuberans (*COL1A1-PDGFB*), one endometrial stromal sarcoma (*YWHAE-NUTM2B*, aka *YWHAE-FAM22B*), one gastrointestinal neuroectodermal tumor (*EWSR1-CREB1*), one inflammatory myofibroblastic sarcoma (*TPM4-ALK*), one inflammatory myofibroblastic tumor (*TFG-ROS1*), 2 myoepitheliomas (one *FUS-NFATC2* and one *TRPS1-PLAG1*), 2 sclerosing epithelioid fibrosarcomas (one *EWSR1-CREB3L2* and one *FUS-CREB3L2*) and one solitary fibrous tumor (*NAB2-STAT6*). In addition, 2/5 tumors negative for *EWSR1* rearrangements according to FISH, turned out to express a *CIC-DUX4* fusion, leading to the diagnosis of *CIC-DUX4* fusion-positive undifferentiated round cell sarcoma (27). In all these cases the identified fusions were confirmed by RT-PCR.

Finally, the series analyzed included also sarcoma variants typically devoid of pathognomonic fusions (e.g., leiomyosarcoma, osteosarcoma). Thus, the negative result of the NGS profiling in these cases may be considered compatible with the pathological diagnosis.

## Discussion

The expression of fusion transcripts characterizes over a third of sarcomas where it may provide diagnostic, prognostic and predictive information. The cooperative effort described in this work was aimed at assessing feasibility, reliability, and applicability of NGS-based approaches for the detection of pathognomonic fusion transcripts in a routine diagnostic setting.

In line with recent reports (12, 19), our study corroborates the robustness of NGS, and in particular of AMP-FPS profiling, for the detection of clinically relevant fusions in sarcomas. On one hand, our analysis emphasizes the worth of implementing this type of approach in routine diagnostics. On the other hand, it underlines the importance of being aware of the actual detection capability of the panel used (genes covered by the assay) in relation to the specific tumor variant under investigation.

Our study demonstrates also the versatility of certain NGS fusion commercial panels to respond to specific diagnostic needs. In fact, the possibility of further implementing commercially available panels by spiking-in probes for genetic targets not included in the standard version of the assay allows to expand its detection capability. Indeed, beside *PAX3*, due to the recent therapeutic successes of NTRK fusions targeting drugs in solid tumors (7, 8), we are in the process of customizing the AMP-FPS panel by including primers for *NTRK1* and *NTRK2* (currently only *NTRK3* is covered by the AMP-FPS assay).

Importantly, in the presence of a negative result, a re-evaluation of RNA and library quality is mandatory as highly degraded RNA and poor quality libraries may affect the sensitivity of the assay. Nonetheless, we found that apparently low quality samples may still be effective for fusion detection. Indeed, a few cases included in this study (cases #9, 31, 37, 47, 57, 60, 80, 126), although not fulfilling all quality criteria, nevertheless yielded a correct fusion call. This indicates that this type of assay may work even in suboptimal conditions.

Finally, when reporting the result of this type of NGS analysis, especially if negative, a statement specifying the characteristics and the limits of the assay employed (type of NGS panel, number of target genes, website of the provider for the list of targeted fusions) and the actual performance of the test according to the manufacturer's standards (fulfillment of quality parameters) should always be included in the pathology report. It is worth reaffirming that the AMP-FPS assay is designed to target the most common breakpoint regions of the genes covered by the assay. Thus, unusual breakpoints may be source of “false negative” results. Moreover, when dealing with sarcoma variants expressing uncommon fusions, the presence of primers for the target genes should be verified prior to setting up the profiling because the lack of appropriate primers will yield a false negative result. The negativity in the AMP-FPS assay of the two *BCOR* rearranged tumors, included in this series, is instructive in this regard.

In the case of a positive result, beside the genes involved in the fusion, the inclusion in the pathology report of details about the fusion variant detected, including reading frame of the chimeric transcript (in frame/out of frame) and exons involved might be useful. This is of particular importance if the fusion protein is potentially actionable and the retention of specific domains in the chimeric protein is crucial for drug sensitivity, as in the case of NTRK fusions (7–9).

## Data Availability Statement

Sequencing data files are available in the NCBI-SRA (http://www.ncbi.nlm.nih.gov/sra) database under the accession number PRJNA608250.

## Ethics Statement

The studies involving human participants were reviewed and approved by Ethic committee Istituto Ortopedico Rizzoli IRCCS, Regina Elena National Cancer Institute IRCCS, Bambino Gesù Children's Hospital IRCCS and by the proper institutional review boards of the CRO Aviano IRCCS National Cancer Institute, Veneto Institute of Oncology (IOV) IRCCS, University of Padua, Candiolo Cancer Institute FPO-IRCCS, Istituto Scientifico Romagnolo per lo Studio e la Cura dei Tumori (IRST) Meldola IRCCS, Istituto Nazionale dei Tumori di Milano Fondazione IRCCS. Written informed consent to participate in this study was provided by the participants' legal guardian/next of kin.

## Author Contributions

RM conceived the work on the behalf of the ACC sarcoma working group. All authors contributed to the generation of molecular profiling data. Each center involved in panel sequencing was responsible for generation, analyses and sharing of data. RF and RM coordinated the collection and integration of data. DR, MB, DB, FG, and BC were in charge of panel comparison. DR, MB, and DB were in charge of second-level bioinformatic analyses. RM and RF wrote the first draft of the manuscript with the support of DR and MB. All authors revised and approved the final version of the manuscript.

### Conflict of Interest

The authors declare that the research was conducted in the absence of any commercial or financial relationships that could be construed as a potential conflict of interest. The handling editor declared a past co-authorship with two of the authors ET and ABu.

## Supplementary Material

Supplemental Table 1 Fusion transcripts called by the Arriba algorithm.

Supplemental Table 2 Fusion transcripts called by the Pizzly algorithm.

Supplemental Table 3 Fusion transcripts called by the STAR-Fusion algorithm.

## Abbreviations

NGS: next generation sequencing
FFPE: Formalin-Fixed Paraffin-Embedded
FISH: fluorescence *in situ* hybridization
RT-PCR: reverse transcriptase-PCR
RT-qPCR: reverse transcriptase-quantitative PCR
IHC: immunohistochemistry
HC: hybrid capture-based panel
AMP-FPS: Anchored Multiplex PCR FusionPlex Sarcoma panel
TS-Fusion: TruSight RNA Fusion panel
TS-PanCancer: TruSight RNA PanCancer panel
