# Corrigendum: Next-Generation Sequencing Approaches for the Identification of Pathognomonic Fusion Transcripts in Sarcomas: The Experience of the Italian ACC Sarcoma Working Group

**DOI:** 10.3389/fonc.2020.00944

**Published:** 2020-06-23

**Authors:** Dominga Racanelli, Monica Brenca, Davide Baldazzi, Frauke Goeman, Beatrice Casini, Biagio De Angelis, Marika Guercio, Giuseppe Maria Milano, Elena Tamborini, Adele Busico, Gianpaolo Dagrada, Cecilia Garofalo, Chiara Caruso, Antonella Brunello, Ymera Pignochino, Enrico Berrino, Giovanni Grignani, Katia Scotlandi, Alessandro Parra, Claudia Maria Hattinger, Toni Ibrahim, Laura Mercatali, Alessandro De Vita, Maria Vincenza Carriero, Matteo Pallocca, Rossella Loria, Renato Covello, Marta Sbaraglia, Angelo Paolo Dei Tos, Rita Falcioni, Roberta Maestro

**Affiliations:** ^1^Unit of Oncogenetics and Functional Oncogenomics, Centro di Riferimento Oncologico di Aviano (CRO Aviano) IRCCS, National Cancer Institute, Aviano, Italy; ^2^Department of Research, Diagnosis and Innovative Technology, IRCCS Regina Elena National Cancer Institute, Rome, Italy; ^3^Department of Onco-Haematology and Cell and Gene Therapy Unit, Bambino Gesù Children's Hospital, IRCCS, Rome, Italy; ^4^Department of Pathology, Fondazione IRCCS Istituto Nazionale dei Tumori, Milan, Italy; ^5^Advanced Translational Research Laboratory, Veneto Institute of Oncology IOV – IRCCS, Padua, Italy; ^6^Medical Oncology 1, Department of Oncology, Veneto Institute of Oncology IOV – IRCCS, Padua, Italy; ^7^Division of Medical Oncology, Candiolo Cancer Institute, FPO-IRCCS, Candiolo, Italy; ^8^Unit of Pathology, Candiolo Cancer Institute FPO-IRCCS, Candiolo, Italy; ^9^Laboratory of Experimental Oncology, IRCCS Istituto Ortopedico Rizzoli, Bologna, Italy; ^10^Osteoncology and Rare Tumors Center, Istituto Scientifico Romagnolo per lo Studio e la Cura dei Tumori (IRST) IRCCS, Meldola, Italy; ^11^Tumor Progression Unit, Department of Experimental Oncology, Istituto Nazionale Tumori Fondazione “G. Pascale” IRCCS, Naples, Italy; ^12^Department of Pathology, Azienda Ospedaliera Universitaria di Padova, Padua, Italy; ^13^Department of Medicine, University of Padua School of Medicine, Padua, Italy

**Keywords:** sarcoma, molecular diagnosis, fusion transcripts, NGS, anchored multiplex PCR, hybrid capture-based panel

In the original article, the Ethics Statement was incomplete as it only included the name of one of the institutions. The corrected statement appears below.

## Ethics Statement

The studies involving human participants were reviewed and approved by Ethic committee Istituto Ortopedico Rizzoli IRCCS, Regina Elena National Cancer Institute IRCCS, Bambino Gesù Children's Hospital IRCCS and by the proper institutional review boards of the CRO Aviano IRCCS National Cancer Institute, Veneto Institute of Oncology (IOV) IRCCS, University of Padua, Candiolo Cancer Institute FPO-IRCCS, Istituto Scientifico Romagnolo per lo Studio e la Cura dei Tumori (IRST) Meldola IRCCS, Istituto Nazionale dei Tumori di Milano Fondazione IRCCS. Written informed consent to participate in this study was provided by the participants' legal guardian/next of kin.

The authors apologize for this error and state that this does not change the scientific conclusions of the article in any way. The original article has been updated.

